# Security Evaluation under Different Exchange Strategies Based on Heterogeneous CPS Model in Interdependent Sensor Networks

**DOI:** 10.3390/s20216123

**Published:** 2020-10-28

**Authors:** Hao Peng, Can Liu, Dandan Zhao, Zhaolong Hu, Jianmin Han

**Affiliations:** College of Mathematics and Computer Science, Zhejiang Normal University, Jinhua 321004, China; hpeng@zjnu.edu.cn (H.P.); liucan@zjnu.edu.cn (C.L.); huzhaolong@zjnu.edu.cn (Z.H.); hanjm@zjnu.cn (J.H.)

**Keywords:** sensor network, interdependent network, cascading failures, swap inter-links strategy, robustness, giant component

## Abstract

In the real Internet of Everything scenario, many large-scale information systems can be converted into interdependent sensor networks, such as smart grids, smart medical systems, and industrial Internet systems. These complex systems usually have multiple interdependent sensor networks. Small faults or failure behaviors between networks may cause serious cascading failure effects of the entire system. Therefore, in this paper, we will focus on the security of interdependent sensor networks. Firstly, by calculating the size of the largest functional component in the entire network, the impact of random attacks on the security of interdependent sensor networks is analyzed. Secondly, it compares and analyzes the impact of cascading failures between interdependent sensor networks under different switching edge strategies. Finally, the simulation results verify the effect of the security of the system under different strategies, and give a better exchange strategy to enhance the security of the system. In addition, the research work in this article can help design how to further optimize the topology of interdependent sensor networks by reducing the impact of cascading failures.

## 1. Introduction

During the advent of the Internet of Things (IoT), the Internet has become widely applied in our society and life. After all deepening and penetration of Internet applications, more and more sensor networks are playing a vital role in medical, industrial control systems, and military fields. Furthermore, we see that due to the limitations of a single network, a single network cannot complete more complex transactions. The sensor network has evolved from a single and small number of nodes to a complex and huge number of nodes network and accelerating to present interdependent sensor networks [1,2].

The sensor networks consist of a data collection unit, a control unit, a communication unit, and a power unit into defined environments that have specific purposes [3,4,5]. The communication unit can exchange data with other systems [6]. The control unit is necessary to interact with the real world and process the data obtained [6,7,8]. The data collection unit is essential for the interdependent network since the data can be linked and importance can be evaluated [9]. In other words, the sensor network is an embedded system that can send and receive data over networks [8]. To model the interdependent sensor networks, we transform the interdependent network into a cyber network and a physical network. This coupled network is named the Cyber-Physical System (CPS). A large number of infrastructure systems evolve into CPS. For instance, all of the smart grid systems, the transportation systems, and the radar systems are regarded as the typical representative of the interdependent CPS [9,10,11,12,13]. If one faulty node or several failed nodes appear in the above-mentioned interdependent system or other important infrastructure systems, this phenomenon may result in the loss of money or even the death of people. In this way, the reliability of the interdependent network is a leading indicator in interdependent systems, which is explored by scholars. Maintaining the normal working of interdependent networks and enhancing the ability of nodes are important. The scholars have found that a small number of failed nodes in an interdependent network could easily lead to severe cascading failures in the entire system [14,15,16].

Over the last decade, extensive research demonstrates many critical properties of the network’s organization, growth, and robustness [14,15,17]. More recently, research on network robustness has been pushed further. Nodes organize networks, but the network does not occur in isolation. Several dependency networks are constructed into one system for one purpose. To complete this purpose, different networks have to apply different sources, which are needed by other networks.

To adapt to society and human development, scholars focus on improving the reliability of social networks. Based on this goal, we model the interdependent CPS as an unweighted and undirected graph. In this graph, the devices are represented in nodes and the relationship of networks’ devices is shown in links. By reconstructing the relationship of different nodes, we achieve the goal of improving the reliability of interdependent networks. The main contributions of our paper are described in the following:

(i) First, we abstract the interdependent networks into various CPS models and attack at a fixed ratio to obtain the influence of different methods on enhancing the robustness of interdependent networks.
(ii) Second, the high betweenness centrality and high eigenvector centrality swapping inter-links strategies have a better performance than other methods in enhancing *G* and $p_{c}$ in all CPS models, respectively.

The outline of this work is as follows: many efforts in improving reliability are shown in Section 2. In Section 3, we describe our models for the CPS. Section 4 performs the processes of six kinds of swapping strategies in detail. Section 5 is the simulation of our models and we analyzed the reasons for the important points. In the end, conclusions are summarized in Section 6.

## 2. Literature Review

The interdependent sensor network is becoming increasingly critical in daily life. Maintaining the reliability of interdependent CPS has become an important research direction. Many scholars have explored this topic from the hardware perspective [18,19,20,21]. In [11], research work shows that the interdependent network will lead to a large number of new dataflows on the Internet of Production (IoP). In [22], scholars study the realization of multi-state channels and the security of symbol energy, and consider their influence on the acceptance threshold of interdependent networks. The method [21,23] is proposed to change the system state by software to ensure that the system remains safe. Except for changing network reliability of hardware and software, a number of researchers have also promoted studying the reliability of the network of interdependence direction from the evaluation of the safety of the network [24]. Machine learning is gradually applied to enhance the reliability of the interdependent network [18,25,26].

The mentioned studies depart the CPS into the physical devices and the cyber components. The above methods are studied depending on the differences in the network’s equipment. Recently, improving the robustness of the interdependent networks by abstracting the system into a graph is another research direction that has received more attention. This approach ignores the differences between devices and treats all devices and components as objects with similar functions. It pays attention to the point-to-point topological relationship between interdependent networks. Because of the specifics of actuation and the physical world reaction, a unique CPS model is infeasible [17]. To solve this problem, Zhang et al. [27] proposed a classification of interdependent networks that is now widely used. In interdependent CPS, batteries and sensors, for example, seem to be physical components. The cyber components include embedded computers and communication networks. To model the relationship of intra-links and inter-links between these two networks, Wang et al. [28] and Derler et al. [29] proposed different algorithms to solve the above-mentioned relationship between the intra-links and inter-links and related problems.

In the above interdependent network graph models, scholars propose various methods to improve the reliability of the CPS. Tu et al. [30] study the robustness of a single network with different values of network centralities. They find the optimal network topology to achieve the best network robustness. They deduce that if one network has better reliability, the values of metrics that they proposed will be different from before. In one network, there are several important nodes and a large number of unimportant nodes. Keeping important nodes in a safe state could guarantee the entire CPS relatively safe [31,32]. However, Nguyen et al. [32] prove that finding critical nodes in a network graph is an NP-hard problem. When one node fails in one network, it can restore to normal working condition by itself without relying on external force [33]. However, this approach will consume huge amounts of money [34]. Refiguring the relationship of links in one network by rewiring links can improve the reliability of interdependent networks [10,35]. However, it is difficult to come true in an existing factual network. This modification will affect the normal operation of existing networks and cause many existing nodes and links to be abandoned. Therefore, this method will lead to a lot of wasting of resources. Adding links in networks was proposed by XingPei Ji et al. [34,36,37]. They discuss the effects of different addition strategies on the reliability of CPS. Through numerous simulations, they find that interdependent networks can maximize reliability by adding intra-links by low inter degree-degree (IDD) values. This will cause many redundant lines and increase overheads. The approach of adjusting the dependency links allocation [38,39] may not increase the costs of building systems. The pros and cons of these mentioned approaches are listed in Table 1.

Swapping the relationship of inter-links does not affect the existing intra-links distribution. Therefore, the topology of the system will not change much. According to the above point of view, swapping inter-links is a better way to enhance system robustness.

## 3. The Model

**Figure 1:**
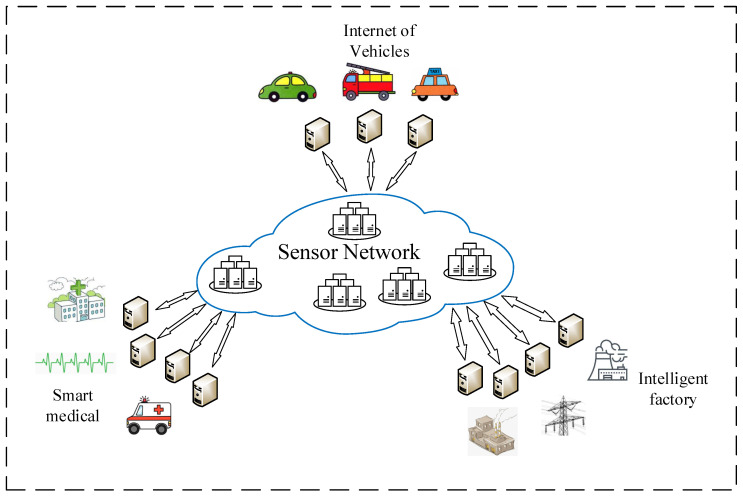
Application scenarios of interdependent sensor networks based on heterogeneous Cyber-Physical System (CPS) architecture.

In reality, interdependent sensor networks have been widely used in our society. All of the Internet of Vehicles, the Intelligent factory, and Smart medical are regarded as sensor networks. They depend on the controlling of a sensor network of the cloud calculating or data transportation. As shown in Figure 1, these application scenarios show the extensiveness of interdependent sensor networks based on heterogeneous CPS architecture, which always consists of all kinds of networks, usually represented by coupled cyber networks and physical networks. In this section, we review different correspondence relationships and processes of cascading failure in interdependent sensor networks. To briefly describe the cascading failure in sensor networks composed of a heterogeneous CPS model, we construct a simple model to perform the cascading failure’s processes in detail.

### 3.1. Interdependent Networks Model

The ‘one-to-one correspondence’ model is suggested by Buldyrev to represent the cascading failure between networks [14]. These coupled two networks are named *A* and *B* in the correspondence model. Based on the ‘one-to-one correspondence’ relationship between networks, each node in network *A* has an inter-link with one node from network *B*, and vice versa. For this reason, the number of nodes in two networks that establish the ‘one-to-one correspondence’ model is the same.

To improve the unity of the ‘one-to-one correspondence’ model, Shao et al. [33,40,41] build a ‘multiple-to-multiple correspondence’ model to imitate the real-world networks. This correspondence relationship implies that a node in network *A* operates depending on several nodes from network *B*, and vice versa. ‘Multiple-to-multiple correspondence’ can better display some characteristics of realistic networks.

‘One-to-multiple correspondence’ model [17,40,42,43] is different to ‘one-to-one correspondence’ and the ‘multiple-to-multiple correspondence’ model. It combines some features of the above two models. Firstly, it increases the singularity of inter-links connection relationships of the ‘one-to-one correspondence’ model. It then improves the overcomplexity of the ‘multiple-to multiple correspondence’ model, which is difficult to explore in studying research. The ‘one-to-multiple correspondence’ model can well simulate the connection of equipment in the smart power grid. One power station can provide power for multiple devices, but one control device only controls one power station. According to this correspondence relationship, the ‘one-to-multiple correspondence’ model is widely used in power grid model simulation.

### 3.2. Cascading Failures Model

Buldyrev et al. study the robustness of the ‘one-to-one correspondence’ model, and they put forward two conditions that must be met at the same time when one node in interdependent networks can normally work [14]:

- This node must belong to the giant component of its network;
- The node must have at least one inter-link from other networks.

They derive the theoretical formula of final nodes number after cascade failures and verify its correctness through experimental simulation:

(1) $$\left\{\begin{array}{cc} x & =g_{A}\left(y\right)p\\ y & =g_{B}\left(x\right)p \end{array}\right.$$

where $g_{A}\left(y\right)\left(g_{B}\left(x\right)\right)$ means the fraction of nodes that belong to the giant component of the network A(B). *p* is the remaining fraction of nodes at the initial attacking stage. They give the derivation formula of the critical value $p_{c}$, which means the maximum value of *p* when CPS is not collapsed completely:

(2) $$1={\left.p^{2}\frac{dg_{A}}{dx}\left[pg_{B}\left(x\right)\right]\frac{dg_{B}}{dx}\left(x\right)\right|}_{x=x_{c},x=p_{c}}$$

Equation (1) in ER networks will transform to:

(3) $$\left\{\begin{array}{cc} x & =p[1-f_{A}]\\ y & =p[1-f_{B}] \end{array}\right.$$

where

(4) $$\left\{\begin{array}{cc} f_{A} & =exp[ay\left(f_{A}-1\right)]\\ f_{A} & =exp[bx\left(f_{B}-1\right)] \end{array}\right.$$

In SF networks, Equation (1) is changing into:

(5) $$x=p\langle k_{A}\rangle {\left[p{\tilde{\kappa }}_{A}\langle k_{B}\rangle {\left({\tilde{\kappa }}_{B}x\right)}^{1/(3-\lambda_{B})}\right]}^{1/(3-\lambda_{A})}$$

${\tilde{\kappa }}_{A}\left({\tilde{\kappa }}_{B}\right)$ is the number of normal working nodes in the network A(B) after the first stage in the cascading failure. $\langle k_{A}\rangle \left(\langle k_{B}\rangle \right)$ is the average degree value of the network A(B). $\lambda_{A}\left(\lambda_{B}\right)$ is the parameter of SF network A(B).

These conditions have been extensively studied and used in network theory. In this study, we use the above conclusions to measure the number of normal working nodes. Failed nodes trigger cascading failures in either network *A* or *B*. The proportion of the initially failed nodes is usually denoted by 1−p. All symbols in the above equations are detailed and represented in Table 2.

We assume that the $\left(1-p\right)|N_{A}|$ number of failed nodes initially appears in the network *A*. The number of nodes in network *A* and *B* is denoted by $N_{A}$ and $N_{B}$. All links of the failed nodes are removed, and network *A* splits into several components. According to the second of the above nodes’ normal working conditions, all nodes in network *A* except the giant component will be removed. In the next stage, network *B* starts to fragment into components since several nodes lost their inter-links from network *A*. A certain amount of nodes separated from the giant component of network *B* are considered as failed nodes and removed all links. Thus, some nodes cannot operate due to a violation of condition I. This will cause the failure to spread from network *B* to network *A*. The nodes that are not following the above two conditions will be removed with their links. The cascading failure occurs recursively in the two networks until the failure stops in one of the following two conditions:

- All nodes are removed, and the interdependent networks are completely collapsing;
- The rest of the nodes both obey conditions I and II. These nodes will not continue to fail nor propagate failures. In this case, the interdependent networks achieve a steady state.

**Figure 2:**
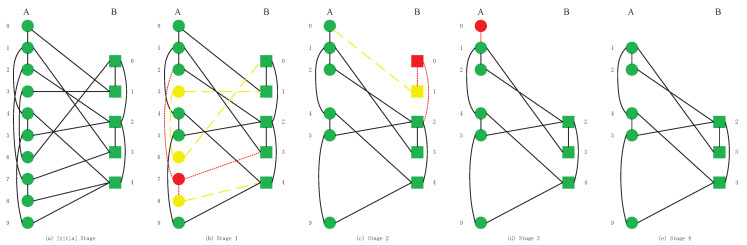
The cascading failure in interdependent networks.

In Figure 2, we give the processes of cascading failures in a simple CPS model. In the interdependent networks model, the ratio of inter-links is 3:1. We intercept the connection of some nodes in the model. We assume that the model links relationships shown in the figure. Ten nodes in network *A* and five nodes in network *B* in the initial stage. Initially, there are ten nodes and five nodes in network *A* and *B*. The random attack upon network *A* causes failure of node $A_{7}$. In the initial stage (Figure 2b), we remove all intra-links and inter-links that are linked to node $A_{7}$ (node is shown in the red circle, and links are shown in solid red lines). Thus, $A_{6}$ and $A_{7}$ (shown in yellow circles) are departed from the giant component of network *A*. In stage 2, $A_{6}$ and $A_{7}$ are removed with all links (shown in yellow dotted lines in (b)). Consequently, two network nodes *B* fail, while node $B_{1}$ (red square) is excluded from the giant component, and $B_{0}$ (yellow square) loses inter-links. In this way, in stage 3, node $B_{0}$, $B_{1}$ and their links are deleted, network *B* fragments into components. Node $A_{0}$ (red circle) fails because it does not have a supporting link from network *B*. In the final stage (Figure 2e), the normal working nodes of this system reach a stable stage and cascading failures will not occur. As the cascading failure stops, there are five nodes in network *A* and three nodes in network *B* can operate normally.

## 4. The Method

**Figure 3:**
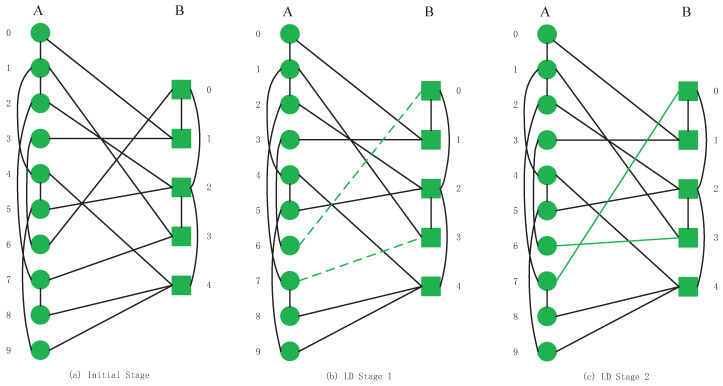
The entire low degree (LD) swapping inter-links strategy processes in interdependent networks.

**Figure 4:**
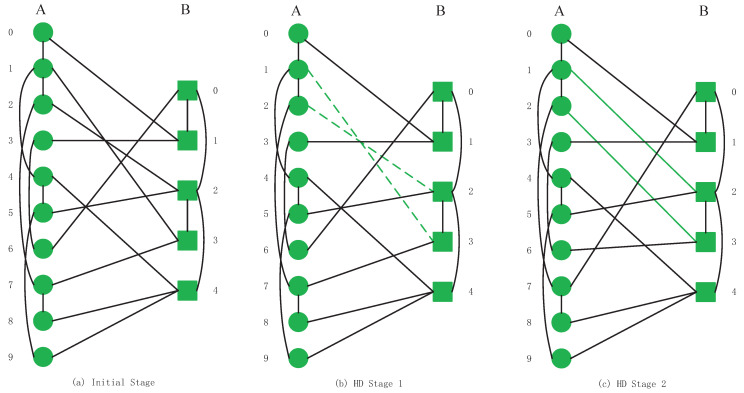
The full high-degree (HD) swapping inter-links strategy processes in interdependent networks.

This section introduces six kinds of swapping inter-links strategies that we apply in our CPS models. To clarify the difference in operation between different strategies, we will exchange inter-links by a low degree swapping strategy and a high degree swapping strategy on the model shown in Figure 2a. After the swapping operations, the inter-links relationships of the CPS will be changed, as shown in Figure 3c and Figure 4c.

At first, we apply NONE as a comparative experiment in our paper. NONE is the behavior of not performing any operations. It means that we use the original network system to suffer random attacks.

### 4.1. Strategy 1: Low Degree (LD)

Degree centrality is one of the most essential and most straightforward metrics to reflect the importance of one node locality in one network [14,36,44]. The LD swapping strategy is calculating all nodes’ degree values and ranking nodes in an increasing order to construct a new interdependent network model. One inter-link is swapped of the two nodes, which have the lowest degree centrality values in these two networks. We must ensure that the total number of inter-links and the number of each nodes’ inter-links in the entire model remain unchanged. For example, node $B_{2}$ has two inter-links with network *A*, and the total number of inter-links is 10 in Figure 2a. We must maintain two inter-links with node $B_{2}$ and the number of inter-links in the entire system is ten after swapping processes. The swapping operations are repeated until the demanding number of nodes’ inter-links are swapped.

In Figure 3a initial stage, we calculate the degree value of a node by determining the number of intra-links within it. Based on the initial system shown in (a), the nodes with the lowest degree value in network *A* are $A_{0}$, $A_{3}$, $A_{6}$, $A_{8}$, $A_{9}$. These nodes all have the same value of degrees, and the value is 1. In network *B*, the lowest degree value nodes are $B_{1}$, $B_{3}$, $B_{4}$ and the degree value is 1. According to the LD strategy requirements, we must ensure that the number of inter-links in nodes does not change. In the model, $A_{0}$ connects $B_{1}$ with an inter-link and $A_{3}$ links $B_{1}$ by one inter-link. All inter-links of $A_{0}$, $A_{3}$ and $B_{1}$ are connected to the nodes with the lowest degree, so no swapping operations are required. $A_{6}$ node depends on $B_{0}$ node while $B_{3}$ depends on nodes $A_{1}$ and $A_{7}$. The degree value of node $A_{7}$ is smaller than the node $A_{1}$. For this reason, we swap inter-links among $A_{6}$, $B_{0}$, $A_{7}$, and $B_{3}$ as indicated in (b) by green dotted lines. The inter-links’ relationship after a successful LD swapping strategy in the system is shown in Figure 3c by green solid lines. After the above operations, we complete the swapping inter-link operation once.

### 4.2. Strategy 2: High Degree (HD)

High degree (HD) swapping strategy is described as the following: calculating all nodes degree values and ranking nodes in decreasing order by degrees. An inter-link is swapped between two nodes, which have the highest degree centrality values in their networks. We ensure that the total number of inter-links and each node’s inter-links in the entire model remain unchanged. The swapping operation is repeated a demanded number of times.

Next, we swap one inter-link in Figure 4a model by HD strategy. The highest degree of network *A* is node $A_{1}$ and network *B* is node $B_{2}$. Therefore, we swap the existing inter-links between $A_{1}$, $B_{3}$, $A_{2}$ and $B_{2}$, as shown in Figure 4b. The relationship of inter-links of the initial system is that $A_{1}$ links $B_{3}$, $A_{2}$ depends on $B_{2}$. It is shown that $A_{1}$ links $B_{3}$ by an inter-link and $B_{2}$ depends on node $A_{2}$ and $A_{5}$. To realize the connection between $A_{1}$ and $B_{2}$, we need to directly select one node between $A_{2}$ and $A_{5}$ as the swapping inter-link choice. The degree values of node $A_{2}$ and $A_{5}$ are the same and we select $A_{5}$ as swapping nodes (we also can choose $A_{2}$ as one selection). We choose these nodes’ inter-links to complete the swapping operation (green dotted lines in (b)). After swapping operations, the system relationship of inter-links is shown in (c). The green solid lines indicate the dependent relationship after the successful swap of inter-links. The CPS model after completing one time of HD swapping operation is shown in Figure 4c.

### 4.3. Strategy 3: Low Betweenness (LB)

Betweenness centrality is a metric to evaluate nodes’ importance by paths [36,45]. The betweenness centrality values of nodes can be calculated by the following equation:

(6) $$B\left(v\right)=\sum_{i\neq j}\frac{\sigma_{ij}\left(v\right)}{\sigma_{ij}}$$

where $\sigma_{ij}$ is the number of the shortest paths going from node *i* to node *j* and $\sigma_{ij}\left(v\right)$ is the number of shortest paths going from node *i* to node *j* through node *v* [36,44,46].

LB swapping strategy is the following: calculating all nodes betweenness centrality values and ranking nodes to increase order with degree values. An inter-link is swapped between two nodes, which have the lowest betweenness centrality values. We have to ensure that the total number of inter-links and the number of each nodes’ inter-links in the entire model remain unchanged. The swapping operation is repeated until the specified number of nodes’ inter-links is swapped.

### 4.4. Strategy 4: High Betweenness (HB)

The HB swapping strategy is described as calculating all nodes betweenness centrality values and ranking nodes in descending order. An inter-link is swapped between two nodes, which have the highest betweenness centrality values. We have to ensure that the total number of inter-links and the number of each nodes’ inter-links in the entire model remain unchanged. The swapping operation is repeated until a demanded number of nodes’ inter-links are swapped.

### 4.5. Strategy 5: Low Eigenvector Centrality (LEC)

The eigenvector centrality is a metric to measure the nodes’ importance, and it is an extension of degree centrality [46]. The eigenvector centrality fully considers both of the importance of nodes’ neighbors and the number of neighbors. If one node’s neighbor is essential, this node will be considered significant, too [44].

To calculate all nodes’ eigenvector centrality values, we need to construct an all nodes’ adjacency matrix *A* and $A_{ij}$ is an element of this matrix. $x_{i}$ is the eigenvector centrality value of node *i*. We set the value of the initial $x_{i}$ as 1. Then we use the initial $x_{i}$ to calculate a new value of $x_{i}^{\prime }$. The value of $x_{i}^{\prime }$ is the sum of the neighbors, eigenvector centrality values of node *i*’s: [44,46]:

(7) $$x_{i}^{\prime }=\kappa_{1}^{-1}\sum_{j}A_{ij}x_{j}$$

where $\kappa_{1}$ is the largest eigenvector value of *A*.

The LEC swapping strategy operates as follows: calculating all node eigenvector centrality values and ranking nodes in increasing order. An inter-link is swapped of the two nodes, which have the lowest eigenvector centrality values. We have to ensure that the total number of inter-links and the number of each nodes’ inter-links in the entire model remain unchanged. The swapping operation is repeated until the demanded number of nodes’ inter-links are swapped.

### 4.6. Strategy 6: High Eigenvector Centrality (HEC)

The HEC swapping strategy operates as follows: calculating all nodes’ eigenvector centrality values and ranking them in descending order by eigenvector centrality values. An inter-link is swapped between two nodes, which hold the highest eigenvector centrality values. We make sure that the total number of inter-links in the entire model and each of the nodes’ inter-links remains unchanged. The swapping operation is repeated until the specified number of nodes’ inter-links are swapped.

## 5. Simulation Results and Analysis

In Section 5, we simulate the interdependent sensor networks models. By modeling the processes of cascading failures in models, we obtain the conclusions of which strategies have the best influences in enhancing interdependent network reliability. In [39], scholars have studied system reliability in ‘one-to-one correspondence’ and ‘one-to-multiple correspondence’ under the HB swapping strategy. Their models are built by BA networks. More swapping strategies are simulated in the ‘one-to-one correspondence’ system in [44]. To study how six kinds of swapping strategies affect the robustness of the ‘one-to-multiple correspondence’ system, we conduct the following simulations.

To get more universal conclusions, we build four kinds of CPS models. Erdös and Rényi constructs the network models ER network. The scale-free network (SF network) is used in our model, too. The average degree is 〈k〉=4 in all networks that we build. The parameter λ=3 in the SF network. According to the current state of an interdependent network such as the smart power grid, we regard ‘one-to-multiple correspondence’ as the dependence relationship of two networks in our simulation models. We set $N_{A}$ and $N_{B}$ as 15,000 and 5000, and the connection ratio of the dependency relationship is 3:1. The graph of the CPS is unweighted and undirected, so the intra-links and inter-links are bidirectional.

Then we use different swapping strategies to change the randomly connected inter-links in these models. Due to constraints such as economic conditions and operational complexity, we define the fraction of swapping inter-links between nodes $f_{N}$ as:

(8) $$f_{N}=\frac{N_{S}^{\prime }}{N_{A}}$$

where $N_{S}^{\prime }$ is the total number of inter-links that are swapped by strategies. $N_{A}$ is the node number of network *A*, which is the same as the number of inter-links.

After swapping inter-links operations, we introduce random attacks into the model as attacks on the network. We implement an accidental removal of the network *A* node with the ratio (1−p) as the failed node under random attacks. To reduce the experimental results’ error, we simulate 20 times for each 1−p under one certain swapping strategy and $f_{N}$. The parameters that we use in our simulation have been detailed and represented in Table 3. We take the average of these results as the final simulation results. We take *G*, which means the proportion of nodes in the giant component, to measure the CPS’s reliability. *G* can be calculated as:

(9) $$G=\frac{N_{A}^{\prime }+N_{B}^{\prime }}{N_{A}+N_{B}}$$

$N_{A}^{\prime }\left(N_{B}^{\prime }\right)$ is the number of normal working nodes at the final steady-state. To measure maximum tolerant ability against random failure, we observe the values of $p_{c}$ in the following figures.

**Figure 5:**
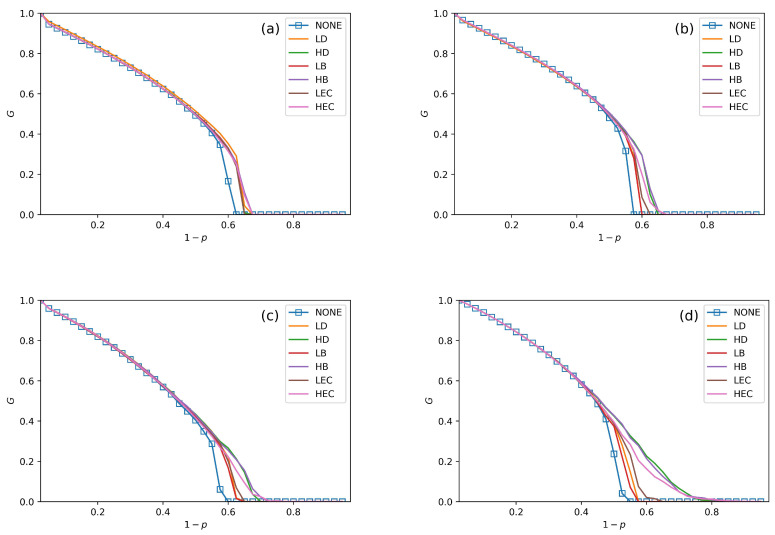
The fraction of function nodes in systems when $f_{N}$ = 30% in ER–ER, ER–SF, SF–ER, and SF–SF systems, which is shown in (**a**–**d**), respectively.

**Figure 6:**
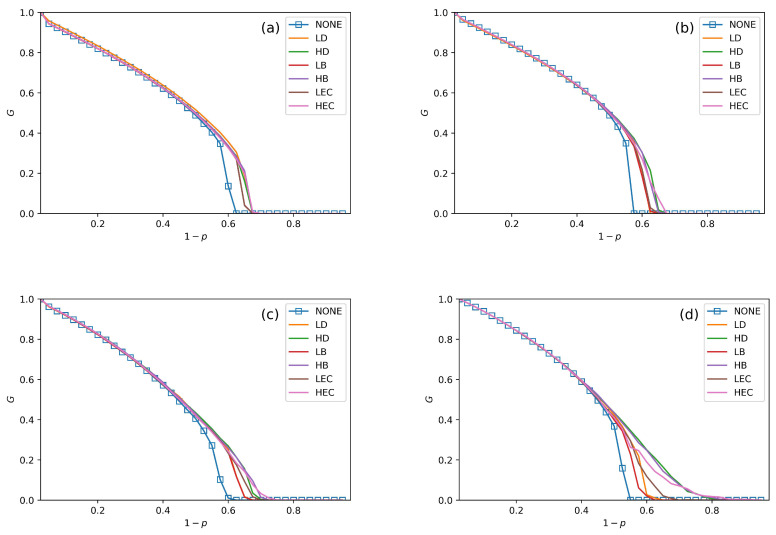
The fraction of function nodes in systems when $f_{N}$ = 50% in ER–ER, ER–SF, SF–ER, and SF–SF systems, which are shown in (**a**–**d**), respectively.

**Figure 7:**
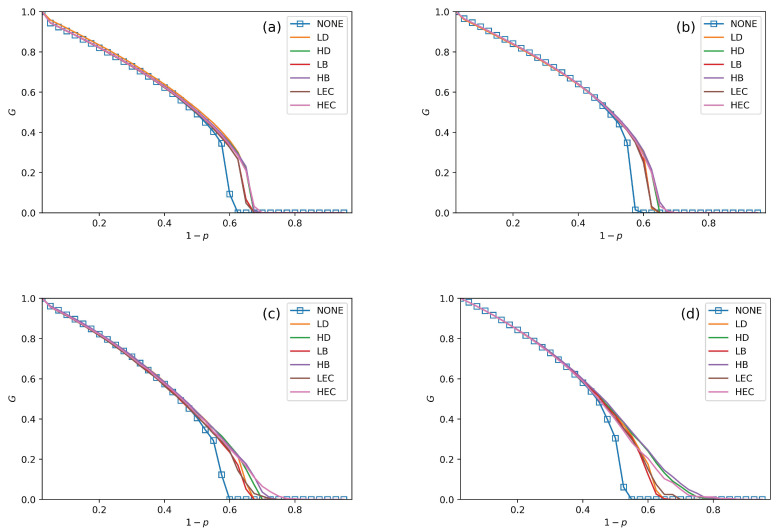
The fraction of function nodes in systems when $f_{N}$ = 70%. (**a**–**d**) are the systems that are being combined with ER–ER, ER–SF, SF–ER, and SF–SF, respectively.

We conduct performance comparisons among the six swapping strategies discussed in Section 4. The values of $f_{N}$ in Figure 5, Figure 6 and Figure 7 are 30%, 50% and 70%. In all figures, we plot the relationship between *G*, $p_{c}$ and 1−p under no swapping operation (NONE) as a contrast experiment for other strategies. From Figure 5, Figure 6 and Figure 7, we can obtain the following situations and get several conclusions:

- All swapping strategies perform better than NONE in improving *G* and $p_{c}$. The values of *G* are clearly bigger in swapping strategies than NONE when 1−p increases. For example, the values of *G* in NONE are lower than the other strategies when (1−p)>0.58 in Figure 5a. When (1−p)>0.5, the value of *G* in NONE is lower than other strategies in Figure 6b. This situation can be observed in all four subfigures in Figure 5, Figure 6 and Figure 7.
  The value of $p_{c}$ is increasing with $f_{N}$ increases. For instance, the values of $p_{c}$ in Figure 5a, Figure 6a and Figure 7a are getting closer and closer to 0.7, and the $p_{c}$ values are closer to 0.75 among Figure 5c, Figure 6c and Figure 7c.
- In Figure 5, Figure 6 and Figure 7, all curves can be divided into three categories. The first is NONE, which shows the worst performance in improving system reliability. The second is swapping inter-links by low centrality values, which are LD, LB, and LEC strategies. Although they show better performance than NONE in enhancing *G* and $p_{c}$, they are not the best choices to achieve more robust systems. The last category is swapping inter-links with high centrality values. High centrality swapping strategies increase the values of *G* and $p_{c}$. We should adopt a high centrality value swapping strategy for improving system reliability. This finding is the same conclusion as in [39,44].
- The sharp drop of *G* gets relief under all swapping strategies. This phenomenon is best reflected in Figure 5d, Figure 6d and Figure 7d. When 1−p gets close to $p_{c}$, the *G* value of NONE is sharply decreased. The stark contrast is the *G* value under the HB strategy in the SF–SF system, which is smoother. This finding means that swapping inter-links in a CPS combined by SF networks is more sensitive in enhancing reliability than combining by ER networks. This conclusion is also found in the ‘one-to-one correspondence’ model [44].
- From all subfigures, we plot in Figure 5, Figure 6 and Figure 7. We conclude that the HB swapping strategy can be the first choice in improving *G* and HEC is the first choice in improving $p_{c}$ values. HB strategy shows the best performance in improving the value of *G*, and the HEC strategy is better in enhancing $p_{c}$ values. The values of $p_{c}$ in Figure 5a–d figures under the HEC strategy are 0.66, 0.69, 0.73 and 0.84, in Figure 6a–d are 0.68, 0.69, 0.75 and 0.85, and in Figure 7a–d are 0.68, 0.7, 0.78 and 0.82. This conclusion is more significant in the SF–SF CPS model. In Figure 5d, the value of $p_{c}$ under HEC is close to 0.8. The value of $p_{c}$ with HEC is more than 0.8 in Figure 6d. This finding is different from [39,44]. We reveal that network construction plays a vital role in system reliability.

## 6. Conclusions and Future Work

To analyze the security of interdependent sensor networks, this paper first constructs a heterogeneous CPS system model with one fixed ratio. On this basis, the security enhancement algorithm of interdependent sensor networks under different swapping link strategies is realized. Next, we analyze the security of interdependent sensor networks by calculating the ratio of the giant component weight of *G* to the maximum resistance to random attacks $p_{c}$ after cascading failures. Finally, through comparative analysis of simulation experiments, better system security can be obtained through a higher central value swapping link method. The experiments results represent the high betweenness centrality swapping strategy is more effective in improving *G*, and the high eigenvector centrality swapping strategy is a better choice in improving $p_{c}$ than other simulation strategies. At the same time, another important conclusion is that the SF network is more sensitive to the security of the system by exchanging internal links than in the ER network.

However, the model proposed in this paper still has some limitations. In the next step, we should build a better, more complex, and interdependent empirical model of sensor networks to simulate actual network systems. In addition, in the next step, we need to find more security enhancement strategies or artificial intelligence optimization algorithms to optimize the security enhancement strategies proposed in this article. 

## Figures and Tables

**Table 1:** Approaches in interdependent systems.

Approaches	Pros	Cons
Protecting critical network nodes	This method has strong pertinence	Finding the critical nodes is an NP-hard problem
Making nodes autonomous	It can make the failure node recover its function and reduce manpower	Expensive; Hard to choosing important nodes
Refiguring the topology of network	It can achieve the purpose of enhancing network reliability	It is not suitable for the existing network
Adding intra-links in systems	Simple; More choices	Increase cost
Adjusting dependency link allocation	The amount that needs to be exchanged is relatively small	The inter-link’s distance between nodes is longer than intra-link

**Table 2:** Key notations in the analysis of cascading failures functions.

Symbol	Meaning
1−p	The fraction of attacked nodes at the first stage
$N_{Ai}$, $N_{Bi}$	The fraction of nodes in the giant component of network *A*, *B* in stage *i*
$N_{Ai}^{\prime }$, $N_{Bi}^{\prime }$	The number of nodes remaining in network *A*, *B* in stage *i*
$\mu_{i}$	The fraction of remaining in network nodes
$\mu_{i}^{\prime }$	The fraction of normal operation nodes in network
$g_{A}$, $g_{B}$	The generating functions of network *A*, *B*
*x*, *y*	The final stage nodes’ number of network *A*, *B*

**Table 3:** The parameters of our simulation models.

Symbol	Values
$N_{A}$	15,000
$N_{B}$	5000
〈k〉	4
λ	3
simulation times of each 1−p	20
